# Dynamic MRI of swallowing: real-time volumetric imaging at 12 frames per second at 3 T

**DOI:** 10.1007/s10334-021-00973-6

**Published:** 2021-11-15

**Authors:** Luuk Voskuilen, Jasper Schoormans, Oliver J. Gurney-Champion, Alfons J. M. Balm, Gustav J. Strijkers, Ludi E. Smeele, Aart J. Nederveen

**Affiliations:** 1 Department of Head and Neck Oncology and Surgery, Netherlands Cancer Institute, Antoni van Leeuwenhoek, Plesmanlaan 121, 1066 CX Amsterdam, The Netherlands; 2 Department of Radiology and Nuclear Medicine, Amsterdam University Medical Centers, University of Amsterdam, Cancer Center Amsterdam, Amsterdam, The Netherlands; 3 Academic Centre for Dentistry Amsterdam and Academic Medical Center, University of Amsterdam and VU University Amsterdam, Amsterdam, The Netherlands; 4 Biomedical Engineering and Physics, Amsterdam University Medical Centers, University of Amsterdam, Amsterdam, The Netherlands; 5 Department of Oral and Maxillofacial Surgery, Amsterdam University Medical Centers, University of Amsterdam, Amsterdam, The Netherlands; 6 Robotics and Mechatronics, faculty of EEMCS, TechMed Center, University of Twente, Enschede, The Netherlands

**Keywords:** Swallowing, Real-time, Stack-of-stars, Golden angle, Compressed sensing

## Abstract

**Objective:**

Dysphagia or difficulty in swallowing is a potentially hazardous clinical problem that needs regular monitoring. Real-time 2D MRI of swallowing is a promising radiation-free alternative to the current clinical standard: videofluoroscopy. However, aspiration may be missed if it occurs outside this single imaged slice. We therefore aimed to image swallowing in 3D real time at 12 frames per second (fps).

**Materials and methods:**

At 3 T, three 3D real-time MRI acquisition approaches were compared to the 2D acquisition: an aligned stack-of-stars (SOS), and a rotated SOS with a golden-angle increment and with a tiny golden-angle increment. The optimal 3D acquisition was determined by computer simulations and phantom scans. Subsequently, five healthy volunteers were scanned and swallowing parameters were measured.

**Results:**

Although the rotated SOS approaches resulted in better image quality in simulations, in practice, the aligned SOS performed best due to the limited number of slices. The four swallowing phases could be distinguished in 3D real-time MRI, even though the spatial blurring was stronger than in 2D. The swallowing parameters were similar between 2 and 3D.

**Conclusion:**

At a spatial resolution of 2-by-2-by-6 mm with seven slices, swallowing can be imaged in 3D real time at a frame rate of 12 fps.

**Supplementary Information:**

The online version contains supplementary material available at 10.1007/s10334-021-00973-6.

## Introduction

Dysphagia or difficulty in swallowing is a potentially hazardous complication of diseases ranging from neurological disorders [1] to head-and-neck cancer [2]. Dysphagia can be classified either as a mechanical obstruction, for example the compression of the pharyngeal tract by a tumour mass, or as a motility disorder, often indicating a neuromuscular disease [3]. Whatever the cause, oropharyngeal dysphagia may have a serious negative impact on the food intake [4], reducing the quality of life [5], and increases the chance of aspiration and subsequent pneumonia [6], which may be life threatening. The swallowing functionality of patients suffering from dysphagia should therefore be monitored regularly.

The most commonly used method for evaluating swallowing functionality is the videofluoroscopic swallowing study (VFSS) [7], also called modified barium swallowing study. While the patient swallows a radiopaque contrast agent, a fluoroscope visualises the oral and pharyngeal phases of swallowing, and any aspiration of the contrast agent. Although the VFSS is the diagnostic test of choice for oropharyngeal dysphagia [3], it is subject to several disadvantages: it has poor soft-tissue contrast and it superimposes anatomical structures making 3D localisation difficult. Moreover, it exposes the patient to ionising radiation, which is especially harmful in persistent dysphagia patients as multiple follow-up consultations are required [8].

Dynamic MRI of swallowing has been proposed as an alternative to the VFSS [9] that is able to visualise the mobility of soft tissues [10]. Until recently, the main disadvantage of dynamic MRI of swallowing had been the low temporal resolution of only several frames per second (fps). However, advances in MRI acceleration, such as compressed sensing [11], have shown that it is feasible to study swallowing with MRI in real time in 2D at 24.3 fps [12], which is a similar frame rate to the VFSS.

Unlike the VFSS, real-time MRI of swallowing is not based on ionising radiation, and provides better soft-tissue contrast. However, as this real-time MRI is mostly acquired only in a single 2D slice, if aspiration occurs outside this imaged midsagittal slice, it will be missed. Such aspiration could be detected on the projection images generated in VFSS, although without a 3D localisation due to the superimposition of the anatomical structures. We can overcome the limitations of the VFSS and conventional 2D real-time MRI by developing real-time 3D MRI for swallowing.

However, with a naïve extension of 2D MRI to 3D real-time MRI, the frame rate would decrease linearly with the number of slices, which may hinder a correct assessment of swallowing. Recently, a frame rate of 15 fps had been shown to be sufficiently high to correctly image swallowing using the VFSS [13]. 3D real-time MRI of swallowing should therefore aim to approach this frame rate of 15 fps as closely as possible.

In this study, we aim to image swallowing in 3D real time at 15 fps. We evaluated three acquisition patterns based on the stack-of-stars (SOS) by computer simulations, and by comparing the image quality provided by these patterns in a static phantom and in healthy volunteers. Finally, we demonstrated the swallowing features that can be visualised with this approach and compare swallowing metrics from this 3D approach to those from the 2D alternative.

## Materials and methods

### Acquisition strategies

For 2D real-time MRI at 3 T, the radial acquisition pattern is often used [12, 14, 15], as it is more resistant to motion artefacts than Cartesian imaging. Furthermore, if subsequent radial k-lines or spokes are rotated by the golden angle (approximately 111.24°), a near-uniform distribution of spokes is ensured, especially if the number of spokes belongs to the Fibonacci sequence [16]. Additionally, it allows an arbitrary time window length and position, which means that a sliding window reconstruction can be used. We therefore aimed to preserve these properties of the 2D radial golden-angle acquisition in a 3D stack-of-stars (SOS) acquisition.

The most straightforward way of extending 2D radial to 3D is the aligned SOS (ASOS), in which the k_z_-direction is fully sampled for each angle [17]. For static SOS imaging, Zhou et al. [18] recently showed that the image quality can be improved by rotating the individual stars over the slice direction (k_z_-direction) in an SOS acquisition using the golden ratio (RSOS-GR). In this implementation, however, the rotation between the stars is dependent on the number of spokes and a sliding window can no longer be used.

In this study, we rotate the stars along the k_z_-direction by the same golden angle that is used to rotate the spokes in the k_x_–k_y_-plane (RSOS-${\psi }_{1})$. The angular increment over the k_z_-direction (inner scan loop) should be continued by the increment in-plane (outer scan loop). Using this continuous golden angle, we hypothesise that the image quality may be improved similar to the work by Zhou et al. [18], while a sliding window reconstruction can also be used.

As the angle increment in the radial golden-angle acquisition is large (111.25°), eddy currents may be induced that distort the images [19]. Eddy currents could therefore negate the image-quality improvement by rotating the stars. Similar to the golden angle, tiny golden angles allow a sliding window reconstruction, while the spokes are distributed evenly [20]. Although the first tiny golden angle (${\psi }_{1}$) is equal to the golden angle, the subsequent tiny golden angles are smaller and thus should induce fewer eddy currents. To ensure an even distribution of spokes in the tiny-golden-angle rotated stack-of-stars (RSOS-${\psi }_{9}$), we chose the tiny golden-angle number equal to the number of samples in the k_z_-direction (${\psi }_{9}\approx 18.71^\circ$).

### Simulations

To compare four sampling strategies (ASOS, RSOS-GR, RSOS-${\psi }_{1}$, and RSOS-${\psi }_{9}$), we determined the incoherence of the PSF similar to the simulations by Zhou et al. [18]. The number of read-out points (256) and the number of slices (nine including oversampling) were the same as those of the phantom and in vivo scans. To obtain the PSF in image space, the spokes were transformed using a non-uniform fast Fourier transform without Toeplitz embedding in BART (version 5.0.0) [21] and MATLAB (R2019a, Mathworks, Natick, MA). Finally, the incoherence of the PSF was quantified by dividing the magnitude of the central peak by the standard deviation of the side lobes.

### Pineapple phantom

Three acquisition patterns (ASOS, RSOS-${\psi }_{1}$, and RSOS-${\psi }_{9}$) were implemented on a 3 T Philips Ingenia scanner (Best, The Netherlands) and evaluated by scanning a pineapple. The RSOS-GR acquisition strategy, for which the spoke angle is dependent on the number of spokes, is not compatible with a sliding window approach, in which the number of spokes may vary, and was therefore not implemented. For each acquisition pattern, a scan was performed for 402 spokes per slice (fully sampled), resulting in a scan time of 1.2 s per slice. First, the number of slices was set to 7, which would be sufficient to cover the oral cavity and oropharynx given the slice thickness of 6 mm. Subsequently, these three acquisition patterns were repeated with 21 slices. The other scan parameters were identical for the three acquisitions: 3D FFE; TR/TE = 3.0/1.26 ms; flip angle: 10°; read-out samples: 256; FOV: matrix size: 128 × 128; voxel size 2 × 2 × 6 mm^3^ (AP × FH × RL); slice oversampling: 1.28 times the number of slices; receiver coils: torso and table coils.

The images of the pineapple phantom were reconstructed using Matlab (R2019a, Mathworks, Natick, MA) and BART (version 5.0.0) [21]. A radial phase shift correction [22] and noise pre-whitening were performed on the spokes before reconstruction. Assuming that sensitivity maps are constant over time, these maps were estimated from low-resolution images reconstructed from all spokes using ESPIRiT [23]. From all 402 spokes per slice, a fully sampled reference image was reconstructed using SENSE [24]. Subsequently, six spokes per slice were binned for each frame, resulting in a frame rate of 6.2 fps for the 7-slice acquisition and 2.1 fps for 21-slice acquisition. The real-time images were reconstructed using compressed sensing with locally low-rank regularisation in plane (with regularisation parameter $\lambda =0.001$) [25] and total variation regularisation over time (with $\lambda =0.005$), which were chosen empirically based on the image quality of the reconstructed images.

To determine the quality of the compressed-sensing reconstruction, we calculated the structural similarity index (SSIM) [26] between the fully sampled ground truth image and the central frame of the accelerated images.

### Healthy volunteers

Five healthy volunteers were included (1 female, mean age 28 years, range 26–29 years), from whom we obtained written informed consent. For this study, we received the appropriate approval from the institutional medical ethical committee. Exclusion criteria were metal braces, dental splints, or any general MRI contraindication. Based on the results from the phantom scans, only the 7-sliced ASOS acquisition was acquired and compared to conventional 2D golden-angle imaging. During these acquisitions, the volunteers were asked to swallow 20 mL of pineapple juice in one swallow, which they administered to themselves with a syringe.

Only the 7-slice acquisition was able to achieve a frame rate of 12 fps, and hence, unlike for the phantom, no acquisitions with 21 slices were made for the volunteers. Additionally, a custom 12-channel flexible surface coil for tongue imaging [14] was used instead of the torso coil. As the two halves of this coil are strapped directly to the cheeks of the volunteers and as it is specifically tuned for tongue imaging, the SNR in the tongue is approximately doubled compared to a conventional neurovascular coil. In addition, the coil features a higher density of coil elements which resulted in better image quality for parallel imaging and compressed-sensing reconstructions [14]. For phantom scans, however, this coil is not suitable as the coil elements have been tuned to the human head. The remaining acquisition parameters were the same as for the pineapple phantom.

The image reconstruction was equal to that of the pineapple phantom, except for two additional steps: a sliding window approach and a flat-field filter. The sliding window was implemented by repeating the reconstruction of the MRI series twice, independently, with half a frame rate shift. Hence, each individual reconstruction was identical to that of the pineapple and simulations, and only after reconstruction, frames were combined to achieve a doubling of the frame rate. The flat-field filter was necessary in vivo to adjust for the local sensitivity changes of the small coil elements used in the dedicated coil array. By dividing a 3D image by that same image convolved with a wide Gaussian kernel, this filter was able to correct for the inhomogeneous image intensity caused by the flexible receiver coil. The flat-field filter also made the comparison possible between the in vivo and phantom scans, which inhomogeneous due to the larger acquisition coils used.

From the 2D and 3D real-time imaging, quantitative parameters of swallowing were measured according to Olthoff et al. [27]: the duration of swallowing, the area of the bolus (in the middle slice for 3D imaging), laryngeal elevation, and contraction of the submental muscles such as the anterior belly of the digastric muscle. For the 3D imaging of swallowing, the bolus volume measurement was added, which was the only measurement based on multiple slices. These parameters were measured by a single rater with 4 years of experience in real-time MRI. The measurements are described in more detail in Online Resource 1.

## Results

**Fig. 1:**
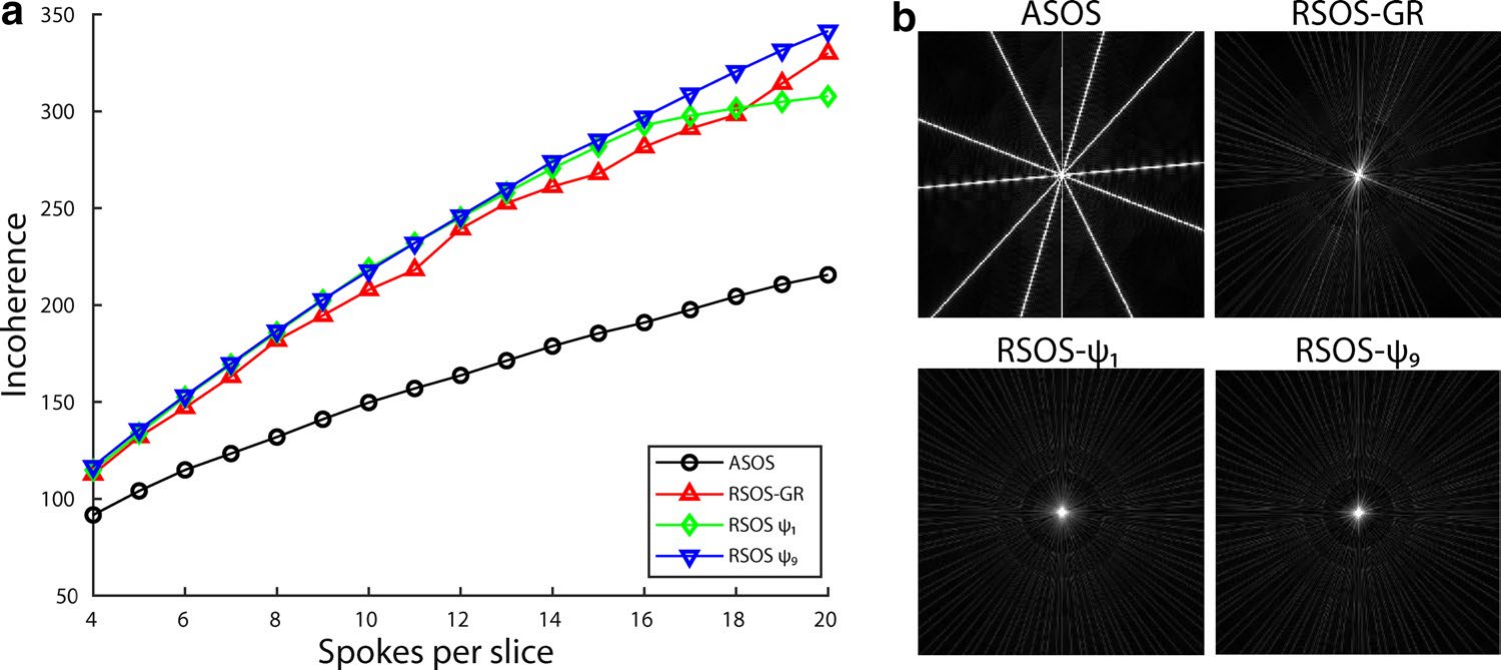
Point-spread function (PSF) simulations for four 3D radial stack-of-stars acquisition patterns. The incoherence was quantified by the ratio of the central peak relative and the standard deviation of the side lobes. In PSFs with higher incoherence, the streaking artefacts were more noise-like, which should benefit a compressed-sensing reconstruction. For the three rotated stack-of-stars (RSOS) acquisition patterns, the incoherence was better than that of the aligned stack-of-stars (ASOS) acquisition (**a**). The difference between the RSOS-GR (Zhou et al. [18]), and the continuous RSOS with the golden angle (RSOS-${\psi }_{1}$) and tiny golden angle (RSOS-${\psi }_{9}$) were small. For six spokes per slice, the central slices of the PSFs for the four acquisition strategies are displayed with the display range from 0 to 10% of the central peak intensity (**b**)

The PSF simulations indicated that the incoherence was higher for all three RSOS approaches (RSOS-GR, RSOS-${\psi }_{1}$, and RSOS-${\psi }_{9}$) than for the aligned SOS, regardless of the number of spokes per slice used (Fig. 1). The difference in incoherence between the three RSOS approaches was marginal, where RSOS-${\psi }_{1}$ and RSOS-${\psi }_{9}$ performed slightly better at fewer than 18 spokes per slice.

**Fig. 2:**
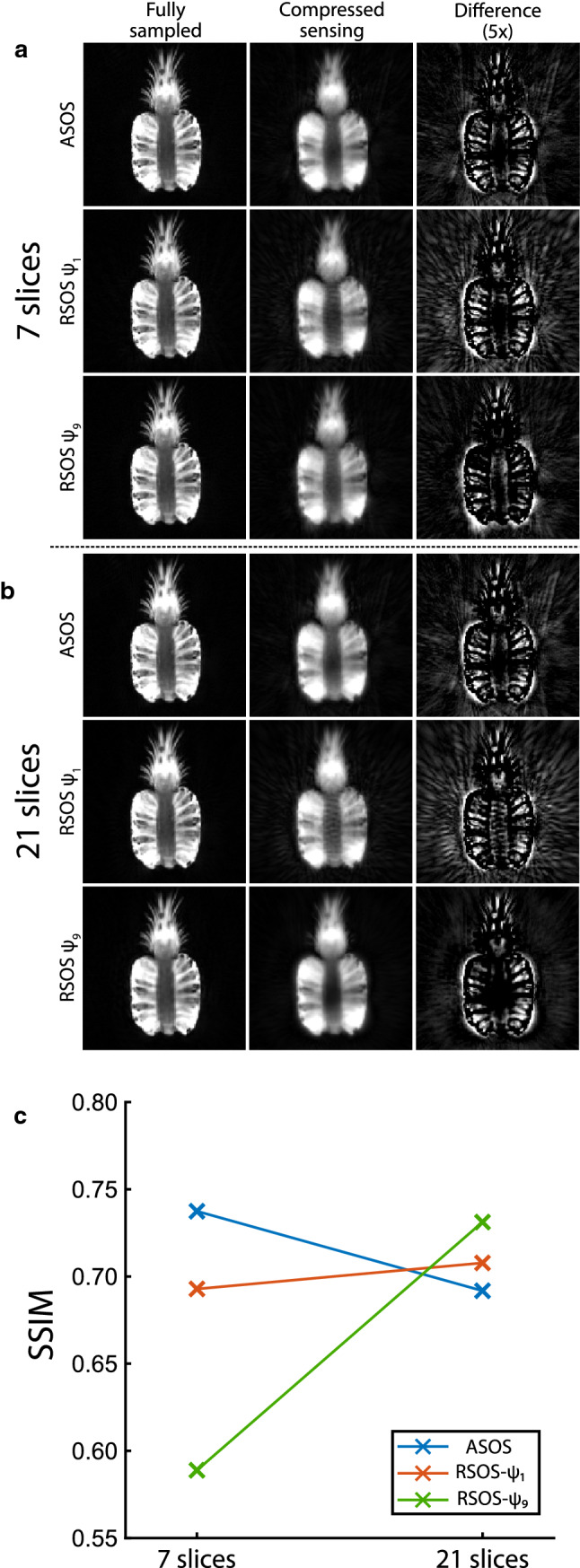
In a pineapple phantom, a fully sampled reconstruction (first column) was compared to a compressed-sensing reconstruction with six spokes per slice (second column). The difference between these reconstructions (third column) is displayed with five times higher window level settings. The three acquisition patterns were acquired with 7 slices (**a**) and 21 slices (**b**), and the structural similarity index (SSIM) between the fully sampled and compressed-sensing reconstruction was calculated (**c**). For both 7 and 21 slices, the RSOS-${\psi }_{1}$ pattern resulted in more radial streaking, and a lower SSIM than for the ASOS. Although the image quality of the ASOS was better than that of the RSOS-${\psi }_{9}$ acquisition for the 7-slice acquisition, the image quality of the RSOS-${\psi }_{9}$ acquisition was better when 21 slices were acquired

For the pineapple phantom, the images reconstructed using compressed sensing displayed a reduction in detail (blurring), which may have been caused by the broader PSF or the compressed-sensing regularisation (Fig. 2). For the acquisitions with 7 slices (Fig. 2a), the SSIM was 0.737 for the ASOS, 0.692 for RSOS-${\psi }_{1}$, and 0.693 for RSOS-${\psi }_{9}$. Contrary to the computer simulations, the image quality of the ASOS acquisition (quantified by the SSIM) was the best of the three, as the RSOS-${\psi }_{1}$ suffered mainly from more radial streaking and the RSOS-${\psi }_{9}$ displayed a stronger halo, which may be best appreciated in the difference images (Fig. 2a).

For the acquisitions of the pineapple phantom with 21 slices (Fig. 2b), the SSIM was 0.708 for the ASOS, 0.589 for RSOS-${\psi }_{1}$, and 0.731 for RSOS-${\psi }_{9}$. The image quality was quantitatively the best for RSOS-${\psi }_{9}$, which was in line with the results from the simulations.

**Fig. 3:**
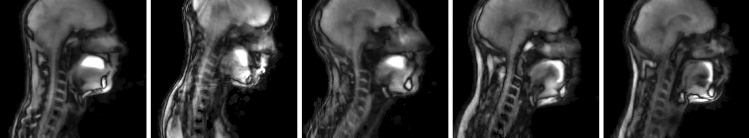
For each of the five healthy volunteers, a frame is displayed just before swallowing pineapple juice. The initial position of the contrast agent (pineapple juice) differed between the volunteers: The first three volunteers have the juice on top of the tongue, while the last two volunteers have the juice in front of the tongue

**Fig. 4:**
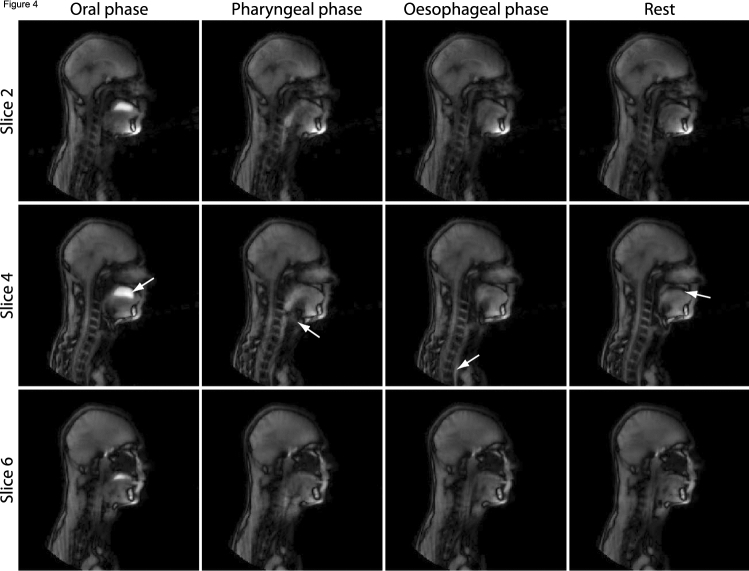
Overview of real-time 3D MRI of swallowing four frames (at equal distance in time) that represent four swallowing phases. Three of the total of seven slices are shown. The contrast agent (pineapple juice) is displayed as a hyperintense fluid in the oral phase (arrow in the first column). Laryngeal elevation and closure by the epiglottis can be appreciated in the pharyngeal phase (arrow in second column). In the oesophageal phase, the contrast agent is located in the oesophagus (arrow third column). Finally, in rest, tongue relaxes and creates space between the palate and itself (arrow last column)

All volunteers were able to swallow the contrast agent (pineapple juice) in a supine position without problems (Fig. 3). The temporal resolution was sufficient such to distinguish the oral, pharyngeal, and the early oesophageal phases of swallowing in all volunteers (Fig. 4, Online Resource 2). In the 3D real-time imaging, the duration of swallowing and the laryngeal elevation were similar to those in 2D (Table 1). The contraction of the submental muscles, however, was smaller for the 3D imaging than for 2D imaging. The bolus volume (only measured in 3D) was larger than the administered volume, most probably due the mixing of the contrast agent with saliva. For 3D imaging, the contraction of the submental muscles is less than for 2D imaging in both studies.

**Table 1:** Quantitative swallowing parameters from 2 and 3D real-time imaging

	This study		Olthoff et al. 2016
Scan mode	3D	2D	2D
Population size	5	5	11
Bolus volume (mL)	20	20	10

	Mean	SD	Mean	SD	Mean	SD
Duration (s)	2.2	0.39	1.9	0.58	4.6	2.0
Bolus (cm^2^)	7.5	2.8	10.5	2.0	3.0	1.4
Bolus (cm^3^)	24.4	5.2	n/a	n/a	n/a	n/a
Laryngeal elevation (mm)	20.4	5.8	20.5	4.5	27	5.0
Contraction of submental muscles (%)	− 25.2	5.0	− 30.6	2.9	− 29	6.0

## Discussion

In this study, we aimed to image swallowing in 3D real time at 3 T. Computer simulations showed that a rotated stack-of-stars (RSOS) approach results in a more incoherent PSF, which should benefit a compressed-sensing reconstruction. For the experiments with the pineapple phantom, the image quality of the RSOS acquisition with the golden angle (RSOS-${\psi }_{1}$) was lower than that of the aligned stack-of-stars (ASOS), which we attribute to increased presence of eddy currents in RSOS-${\psi }_{1}$. A tiny golden-angle approach (RSOS-${\psi }_{9}$) reduced these distortions. However, the image quality of the RSOS-${\psi }_{9}$ approach was only superior to that of the ASOS acquisitions if a sufficient number of slices were acquired. Using the ASOS approach, we were able to image swallowing in 3D real time with a sufficient spatial and temporal resolution to distinguish several swallowing phases and to derive quantitative swallowing parameters: duration, laryngeal elevation, bolus area and volume, and contraction of the submental muscles.

Previously, Zhou et al. [18] found that an RSOS acquisition reduced streaking artefacts. In computer simulations, the incoherence produced by their RSOS-GR approach was higher than that produced by the ASOS; a result confirmed in our study. In this study, we found that a sufficiently high number of slices should be used to benefit from an RSOS approach, and that a tiny golden angle may further improve image quality by reducing eddy-current distortions.

Although we also intended to apply an RSOS approach to our volunteer scans, we observed an unexpected requirement for such an approach. Namely, the phantom scans show that a sufficiently high number of slices are required to benefit from an RSOS approach. This effect may be explained in the following way: If a small number of slices is chosen, the sparsity over slice direction is bad, i.e., there are few zero-valued elements along this dimension in the transformed domain. Therefore, the compressed-sensing reconstruction benefits little from the 3D acquisition. However, as we aimed for a frame rate of 15 fps, which was incompatible with a high number of slices, we ultimately chose for the conventional ASOS approach with seven slices.

Even though the number of slices was reduced to 7, we still only reached a temporal resolution of 12 fps. We decided not to increase the frame rate further, as it would reduce the image quality too much. To push the frame rate to our aim of 15 fps, more time-efficient read-outs could be used.

There are several alternative approaches to real-time MRI that we did not explore in this work. One could use a stack-of-spiral approach to achieve higher frame rate, as spiral trajectories cover more k-space per read-out than radial trajectories and therefore are more time-efficient. Although spiral read-outs can be more challenging than radial read-outs, spiral approaches have already been applied successfully for real-time 3D imaging of speech [28, 29]. It would be interesting to see whether these approaches can be adapted for imaging of swallowing to achieve a frame rate of at least 15 fps.

Although a spiral approach is already being successfully applied to real-time 3D imaging of speech at 1.5 T [28], B_0_-inhomogeneities are more pronounced due to the higher magnetic field at 3 T. Simply acquiring an additional off-resonance field map before the real-time scan may be used to correct for off-resonance artefacts, but it does not contain time-resolved information. Recently, Lim et al. described a method for dynamic off-resonance correction for speech imaging [29] that could help bring spiral imaging to MRI of swallowing.

In addition to rotating in the k_x_–k_y_-plane, the spokes may also be rotated in the k_z_-direction, a so-called koosh-ball trajectory. This acquisition pattern is a useful alternative, especially for isometric field-of-views (FOVs). However, the koosh-ball trajectory requires more spokes to be acquired to meet the Nyquist criterion than an RSOS acquisition would. To account for this, Burdumy et al. [30] implemented an RSOS acquisition pattern, in which the number of spokes per slice was also reduced as the distance from the k-space center increased. Alternatively to 3D MRI, 2D images may be acquired along three orthogonal orientations [31], which already provides a better overview of the swallowing than a single 2D acquisition. However, this imaging approach required multiple swallows, which are difficult to exactly reproduce leading to inconsistencies across slices.

Finally, Fu et al. [32] developed a low-rank method for speech imaging in which a common temporal–spatial subspace is determined from data over repeated talking. If this method could be adopted for imaging of swallowing, this could allow for a substantially higher resolution and frame rate. However, this method would require repeated swallowing, which in its current format (over 7 min of repeated speech) would be physiologically unsafe when applied to swallowing. How these methods perform compared to our SOS approaches should be investigated in future work.

The current reconstruction pipeline is an important hurdle for clinical implementation of the real-time 3D MRI of swallowing. As the whole 4D volume is reconstructed at once, it requires lengthy off-line reconstruction on a high-performance computer (in our case nearly 3 h using four CPUs). If the inverse Fourier transform is first applied along the k_z_-direction, the reconstruction can be considerably accelerated by reconstructing the slices separately in parallel, but this is only an option for the ASOS acquisition.

For 2D and 3D real-time imaging, we determined several parameters to quantify the swallowing movement, which could be compared to previous work by Olthoff et al. [27]. In their study, the duration of swallowing is much longer (mean of 4.6 s), as our definition of a swallowing movement was stricter, meaning that in our case, only a single swallow was included. Between our 2D and 3D scans, the mean laryngeal elevation was nearly equal, but lower than that of Olthoff et al. which may have been caused by inter-rater variability or the smaller fluid bolus administered in their study. Finally, the mean contraction of the submental muscles in 2D is approximately equal to that of Olthoff et al., but for our 3D acquisitions, this contraction is slightly lower. We attribute this decrease to stronger blurring that is present in 3D images compared to the 2D acquisition.

With the exception of the bolus volume, none of the measurements took full advantage of the 3D imaging, as we only intended to determine that there are only minor differences between the 2D acquisition and the 3D acquisition. To better utilise the 3D acquisition, new parameters should be developed. As we are missing patient data in this work, and as there is no similar 3D imaging modality from which metrics may be derived, we decided that the development of new metrics was out of the scope of this work.

Although we were able to perform 3D real-time MRI of swallowing, the main limitation of this study is that we did not compare this technique with the current gold standard for grading dysphagia, VFSS. In the case of inclusion body myositis, 2D real-time MRI has been shown to be able to identify the cause of dysphagia as well as VFSS [15]. However, whether the spatial or temporal resolution of the 3D real-time MRI is sufficient for the detection of aspiration or bolus retention still has to be examined. Comparing VFSS to 3D real-time MRI, we would expect VFSS to provide a better temporal resolution and to be cheaper. In contrast, 3D real-time MRI does not require ionising radiation and provides better soft-tissue contrast, thereby allowing the analysis of swallowing beyond grading the aspiration. A disadvantage may be that MRI scans are generally performed in a supine or prone position, while the natural position for swallowing is upright. Further research should prove whether the position during scanning affects swallowing and thus the grading of dysphagia.

In conclusion, we were able to image and quantify swallowing in 3D real time using MRI at 3 T. We evaluated three SOS acquisition patterns. Although computer simulations showed that the RSOS acquisitions produced a more incoherent PSF that should provide better image quality, the image quality was reduced in RSOS acquisitions probably due to eddy currents. Eddy currents were mitigated by a tiny golden-angle radial k-space filling, which resulted in similar image quality to the aligned SOS. Using this SOS approach, we achieved the imaging of swallowing in 3D with 12 fps, visualising several swallowing phases. We demonstrated that real-time 3D MRI is a potential radiation-free alternative to the VFSS, which can also visualise soft tissues and localise the origin of swallowing problems in 3D.

## Supplementary Information

Below is the link to the electronic supplementary material.

Supplementary file1 ESM1: The definition of the quantitative swallowing metrics visualised on a sagittal slice of the 3D real-time MRI of swallowing. The bolus area and volume were measured by delineating the hyperintense pineapple juice on the image during rest (a). The length of the submental muscles was defined as the distance from the right edge of the mandible to the left edge of the hyoid bone. The contraction of the submental muscles was calculated as the decrease in length during the swallow (b) relative to the length before swallowing (a). The laryngeal elevation was measured as the largest distance between the position of the most inferior point of the thyroid cartilage before swallowing and it position during swallowing. The duration was calculated by counting the frames from the last stationary motion state until the oral tract returned to this state. Eventual additional swallows to completely clear the oral or pharyngeal cavities were not included in the duration.(PNG 1165 KB)

Supplementary file2 ESM2: Visualisation of the full real-time 3D MRI movie of a single swallowing movement. Subsequent slices are placed in reading order: from left to right, up to down. As this is the same movie as visualised in figure 6, the same swallowing phases can be distinguished. (MP4 1291 KB)

## References

[1] Kertscher B, Speyer R, Palmieri M, Plant C (2014). Bedside screening to detect oropharyngeal dysphagia in patients with neurological disorders: an updated systematic review. Dysphagia.

[2] Manikantan K, Khode S, Sayed SI, Roe J, Nutting CM, Rhys-Evans P, Harrington KJ, Kazi R (2009). Dysphagia in head and neck cancer. Cancer Treat Rev.

[3] Jalil AAA, Katzka DA, Castell DO (2015). Approach to the patient with dysphagia. Am J Med.

[4] Mercadante S, Aielli F, Adile C, Ferrera P, Valle A, Fusco F, Caruselli A, Cartoni C, Massimo P, Masedu F, Valenti M, Porzio G (2015). Prevalence of oral mucositis, dry mouth, and dysphagia in advanced cancer patients. Support Care Cancer.

[5] Nguyen NP, Frank C, Moltz CC, Vos P, Smith HJ, Karlsson U, Dutta S, Midyett A, Barloon J, Sallah S (2005). Impact of dysphagia on quality of life after treatment of head-and-neck cancer. Int J Radiat Oncol Biol Phys.

[6] Eisbruch A, Lyden T, Bradford CR, Dawson LA, Haxer MJ, Miller AE, Teknos TN, Chepeha DB, Hogikyan ND, Terrell JE, Wolf GT (2002). Objective assessment of swallowing dysfunction and aspiration after radiation concurrent with chemotherapy for head-and-neck cancer. Int J Radiat Oncol Biol Phys.

[7] Murphy BA, Gilbert J (2009). Dysphagia in head and neck cancer patients treated with radiation: assessment, sequelae, and rehabilitation. Semin Radiat Oncol.

[8] Szczesniak MM, Maclean J, Zhang T, Graham PH, Cook IJ (2014). Persistent dysphagia after head and neck radiotherapy: a common and under-reported complication with significant effect on non-cancer-related mortality. Clin Oncol.

[9] Olthoff A, Carstens PO, Zhang S, von Fintel E, Friede T, Lotz J, Frahm J, Schmidt J (2016) Evaluation of dysphagia by novel real-time magnetic resonance imaging. Neurology 87(20):2132–2138 10.1212/WNL.0000000000003337 27770070

[10] Kreeft AM, Rasch CRN, Muller SH, Pameijer FA, Hallo E, Balm AJM (2012). Cine MRI of swallowing in patients with advanced oral or oropharyngeal carcinoma: a feasibility study. Eur Arch Oto-Rhino-Laryngol.

[11] Lustig M, Donoho DL, Santos JM, Pauly JM (2008). Compressed sensing MRI. IEEE Signal Process Mag.

[12] Zhang S, Olthoff A, Frahm J (2012). Real-time magnetic resonance imaging of normal swallowing. J Magn Reson Imaging.

[13] Layly J, Marmouset F, Chassagnon G, Bertrand P, Sirinelli D, Cottier JP, Morel B (2019). Can we reduce frame rate to 15 images per second in pediatric videofluoroscopic swallow studies?. Dysphagia.

[14] Voskuilen L, de Heer P, van der Molen L, Balm AJM, van der Heijden F, Strijkers GJ, Smeele LE, Nederveen AJ (2020) A 12-channel flexible receiver coil for accelerated tongue imaging. Magn Reson Mater Phys Biol Med 33(4):581–590 10.1007/s10334-019-00824-5 PMC7351800 31950389

[15] Carstens P-O, Zhang S, Olthoff A, Bremen E, Lotz J, Frahm J, Schmidt J (2015). Evaluation of dysphagia in inclusion body myositis by novel real-time MRI. Eur J Neurol.

[16] Winkelmann S, Schaeffter T, Koehler T, Eggers H, Doessel O (2007). An optimal radial profile order based on the golden ratio for time-resolved MRI. IEEE Trans Med Imaging.

[17] Feng L, Grimm R, Block KT, Chandarana H, Kim S, Xu J, Axel L, Sodickson DK, Otazo R (2014). Golden-angle radial sparse parallel MRI: combination of compressed sensing, parallel imaging, and golden-angle radial sampling for fast and flexible dynamic volumetric MRI. Magn Reson Med.

[18] Zhou Z, Han F, Yan L, Wang DJJJ, Hu P (2017). Golden-ratio rotated stack-of-stars acquisition for improved volumetric MRI. Magn Reson Med.

[19] Wundrak S, Paul J, Ulrici J, Hell E, Geibel MA, Bernhardt P, Rottbauer W, Rasche V (2016). Golden ratio sparse MRI using tiny golden angles. Magn Reson Med.

[20] Wundrak S, Paul J, Ulrici J, Hell E, Rasche V (2015). A small surrogate for the golden angle in time-resolved radial MRI based on generalized fibonacci sequences. IEEE Trans Med Imaging.

[21] Uecker M, Ong F, Tamir JI, Bahri D, Virtue P, Cheng JY, Zhang T, Lustig M (2015) Berkeley advanced reconstruction toolbox. Proc Intl Soc Mag Reson Med. 10.5281/zenodo.592960

[22] Buonincontri G, Methner C, Krieg T, Carpenter TA, Sawiak SJ (2014). Trajectory correction for free-breathing radial cine MRI. Magn Reson Imaging.

[23] Uecker M, Lai P, Murphy MJ, Virtue P, Elad M, Pauly JM, Vasanawala SS, Lustig M (2014). ESPIRiT—an eigenvalue approach to autocalibrating parallel MRI: where SENSE meets GRAPPA. Magn Reson Med.

[24] Pruessmann KP, Weiger M, Scheidegger MB, Boesiger P (1999). SENSE: sensitivity encoding for fast MRI. Magn Reson Med.

[25] Ong F, Lustig M (2016). Beyond low rank+ sparse: multi-scale low rank matrix decomposition. 2016 IEEE Int Conf Acoust Speech Signal Process.

[26] Wang Z, Bovik AC, Sheikh HR, Simoncelli EP (2004). Image quality assessment: from error visibility to structural similarity. IEEE Trans image Process.

[27] Olthoff A, Joseph AA, Weidenmüller M, Riley B, Frahm J (2016). Real-time MRI of swallowing: intraoral pressure reduction supports larynx elevation. NMR Biomed.

[28] Lim Y, Zhu Y, Lingala SG, Byrd D, Narayanan S, Nayak KS (2018). 3D dynamic MRI of the vocal tract during natural speech. Magn Reson Med.

[29] Lim Y, Goud S, Shrikanth L, Krishna SN, Lingala SG, Narayanan SS, Nayak KS (2019). Dynamic off-resonance correction for spiral real-time MRI of speech. Magn Reson Med.

[30] Burdumy M, Traser L, Burk F, Richter B, Echternach M, Korvink JG, Hennig J, Zaitsev M (2016). One-second MRI of a three-dimensional vocal tract to measure dynamic articulator modifications. J Magn Reson Imaging.

[31] Olthoff A, Zhang S, Schweizer R, Frahm J (2014). On the physiology of normal swallowing as revealed by magnetic resonance imaging in real time. Gastroenterol Res Pract.

[32] Fu M, Barlaz MS, Holtrop JL, Perry JL, Kuehn DP, Shosted RK, Liang ZP, Sutton BP (2017). High-frame-rate full-vocal-tract 3D dynamic speech imaging. Magn Reson Med.

