# Supplementary figures and images for: Dynamic MRI of swallowing: real-time volumetric imaging at 12 frames per second at 3 T
Source: MAGMA. 2021 Nov 15;35(3):411–9. doi: 10.1007/s10334-021-00973-6 (PMC9188511; doi:10.1007/s10334-021-00973-6)

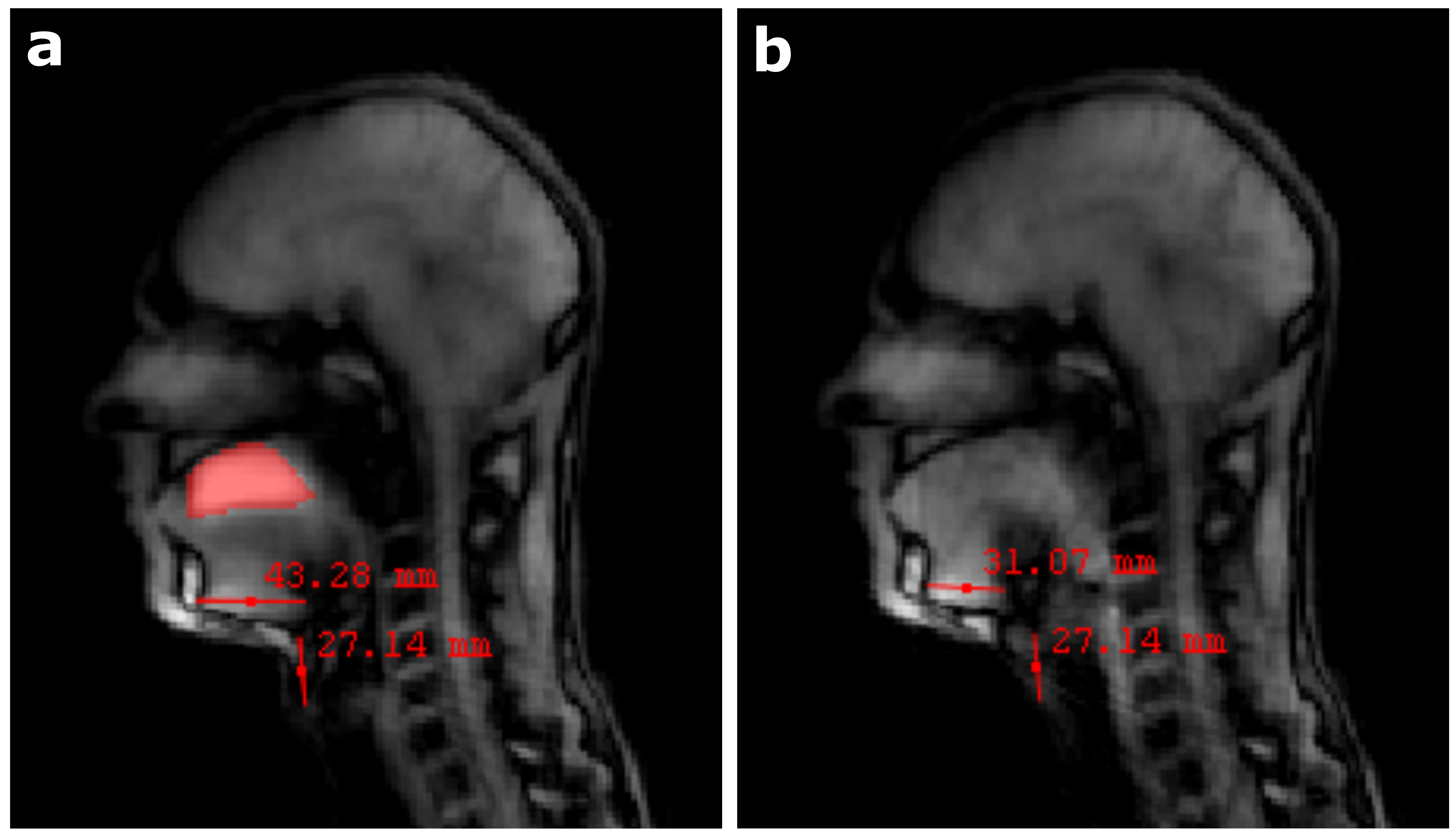

Supplement: Supplementary file 1 — Supplementary file1 ESM1: The definition of the quantitative swallowing metrics visualised on a sagittal slice of the 3D real-time MRI of swallowing. The bolus area and volume were measured by delineating the hyperintense pineapple juice on the image during rest (a). The length of the submental muscles was defined as the distance from the right edge of the mandible to the left edge of the hyoid bone. The contraction of the submental muscles was calculated as the decrease in length during the swallow (b) relative to the length before swallowing (a). The laryngeal elevation was measured as the largest distance between the position of the most inferior point of the thyroid cartilage before swallowing and it position during swallowing. The duration was calculated by counting the frames from the last stationary motion state until the oral tract returned to this state. Eventual additional swallows to completely clear the oral or pharyngeal cavities were not included in the duration.(PNG 1165 KB) [file 10334_2021_973_MOESM1_ESM.png]
